# Postoperative infection following volar locking plate fixation of closed distal radius fractures: a retrospective cohort study of infection rates and antibiotic prophylaxis practice

**DOI:** 10.1186/s12891-026-10521-9

**Published:** 2026-10-03

**Authors:** Daniel Muder

**Affiliations:** 1 Department of Surgical Sciences/Orthopaedics & Hand Surgery, Uppsala University, Entrance 70, Uppsala, SE-75185 Sweden; 2 Center for Clinical Research Dalarna, Uppsala University, Nissers väg 3, Falun, SE-79182 Sweden

**Keywords:** Distal radius fracture, Volar locking plate, Antibiotic prophylaxis, Sunfergical site iction, Fracture surgery

## Abstract

**Background:**

Distal radius fractures are among the most common surgically treated upper-extremity fractures, and volar locking plate fixation is the predominant operative technique. Although perioperative antibiotic prophylaxis is widely used in orthopaedic surgery, procedure-specific evidence for closed distal radius fractures remains limited. Contemporary data regarding postoperative infection rates and antibiotic prophylaxis practice following volar locking plate fixation are scarce. The aim of this study was to describe postoperative infection rates following volar locking plate fixation of closed distal radius fractures and to report contemporary patterns of perioperative antibiotic prophylaxis use in routine clinical practice.

**Methods:**

This retrospective cohort study included consecutive adult patients undergoing volar locking plate fixation for closed distal radius fractures at a single center between January 2022 and December 2024. Patients were grouped according to receipt of perioperative antibiotic prophylaxis, reflecting routine clinical practice rather than predefined treatment allocation. The primary outcome was postoperative surgical site infection within one year of surgery. Secondary analyses described antibiotic prophylaxis use and patient, surgical, and surgeon-related characteristics.

**Results:**

A total of 474 consecutive patients were included, of whom 237 received perioperative antibiotic prophylaxis and 237 did not. One postoperative surgical site infection occurred, corresponding to an overall infection rate of 0.2% (95% confidence interval [CI] 0.0–1.2). The infection occurred in a patient who did not receive antibiotic prophylaxis, was superficial, and resolved following treatment with oral antibiotics. No deep infections were observed. Substantial variation in prophylaxis use was observed among surgeons despite uniformly low infection rates across the cohort.

**Conclusions:**

Postoperative infection following volar locking plate fixation of closed distal radius fractures was rare in this contemporary cohort. The findings provide descriptive data on postoperative infection rates and perioperative antibiotic prophylaxis practice in routine clinical care. Although the study was not powered to evaluate the effectiveness of antibiotic prophylaxis, the observed low infection burden may help inform future research on infection prevention and antibiotic stewardship in distal radius fracture surgery.

**Level of Evidence:**

III.

**Clinical trial number:**

Not applicable

## Introduction

Distal radius fractures (DRFs) are among the most common surgically treated upper extremity fractures and are managed by orthopedic, plastic, and hand surgeons in a wide range of practice settings. The incidence is approximately 280–320 per 100,000 person-years in Sweden [1, 2]. Around 20–25% of these fractures are treated operatively due to instability, displacement or fracture complexity [3, 4]. Operative management has increased over recent decades, with volar locking plate (VLP) fixation now representing the predominant surgical technique [5, 6].

Surgical site infection (SSI) is an uncommon but potentially serious complication following fracture surgery. SSIs may lead to prolonged treatment, reoperation and increased healthcare costs [7, 8]. Preoperative antibiotic prophylaxis is commonly used to reduce infection risk, but its effectiveness depends on procedure type, patient characteristics and baseline infection risk [9].

There is strong evidence supporting antibiotic prophylaxis in several orthopaedic procedures, including open fractures and arthroplasty [10, 11]. However, evidence supporting routine prophylaxis in closed DRF surgery is limited. National guidelines, including those from the National Council on Health Technology Assessment, primarily recommend prophylaxis for high-risk procedures such as open fractures, while practice in closed DRFs varies considerably [12].

Previous studies investigating antibiotic prophylaxis in hand and wrist fracture surgery have largely focused on percutaneous fixation with Kirschner wires and have not demonstrated a clear benefit of routine prophylaxis [13–15]. To date, data specifically addressing antibiotic prophylaxis in volar locking plate fixation of closed distal radius fractures remain scarce.

Given the high incidence of distal radius fractures, the widespread use of volar locking plate fixation, and the continued emphasis on antimicrobial stewardship, contemporary data on postoperative infection rates and perioperative antibiotic prophylaxis practice are warranted. Understanding infection burden and current practice patterns may help inform future research and guideline development in this common orthopaedic procedure. Even a single unnecessary perioperative dose may contribute to antimicrobial resistance, adverse drug reactions, and increased healthcare costs at a population level. Although the individual risk associated with a single prophylactic dose is low, widespread routine use in procedures with very low baseline infection risk warrants evaluation. Considerable variation in prophylactic antibiotic use exists in clinical practice despite limited procedure-specific evidence for closed distal radius fracture surgery. The aim of this study was to describe postoperative infection rates following volar locking plate fixation of closed distal radius fractures and to report contemporary patterns of perioperative antibiotic prophylaxis use in routine clinical practice.

## Methods

### Study design and patient selection

This retrospective cohort study was conducted at a regional hospital. All eligible adult patients undergoing volar locking plate fixation for closed distal radius fractures between January 2022 and December 2024 were consecutively included. The distribution of patients between groups reflected variation in surgeon preference regarding perioperative antibiotic prophylaxis in routine clinical practice rather than predefined allocation, matching, or protocol-driven treatment. Adult patients (≥ 18 years) who underwent operative treatment for closed distal radius fractures using volar locking plates between January 2022 and December 2024 were initially identified using ICD-10 diagnosis and procedure codes S52.5 and NSJ/NDJ 69. Eligibility was subsequently confirmed by review of the operative reports and postoperative radiographs to ensure that all included patients underwent fixation with a volar locking plate according to the predefined inclusion criteria. Inclusion and exclusion criteria are presented in Table 1. Patients treated with additional procedures, open fractures or incomplete follow-up due to cross-county or international care were excluded.

**Fig. 1:**
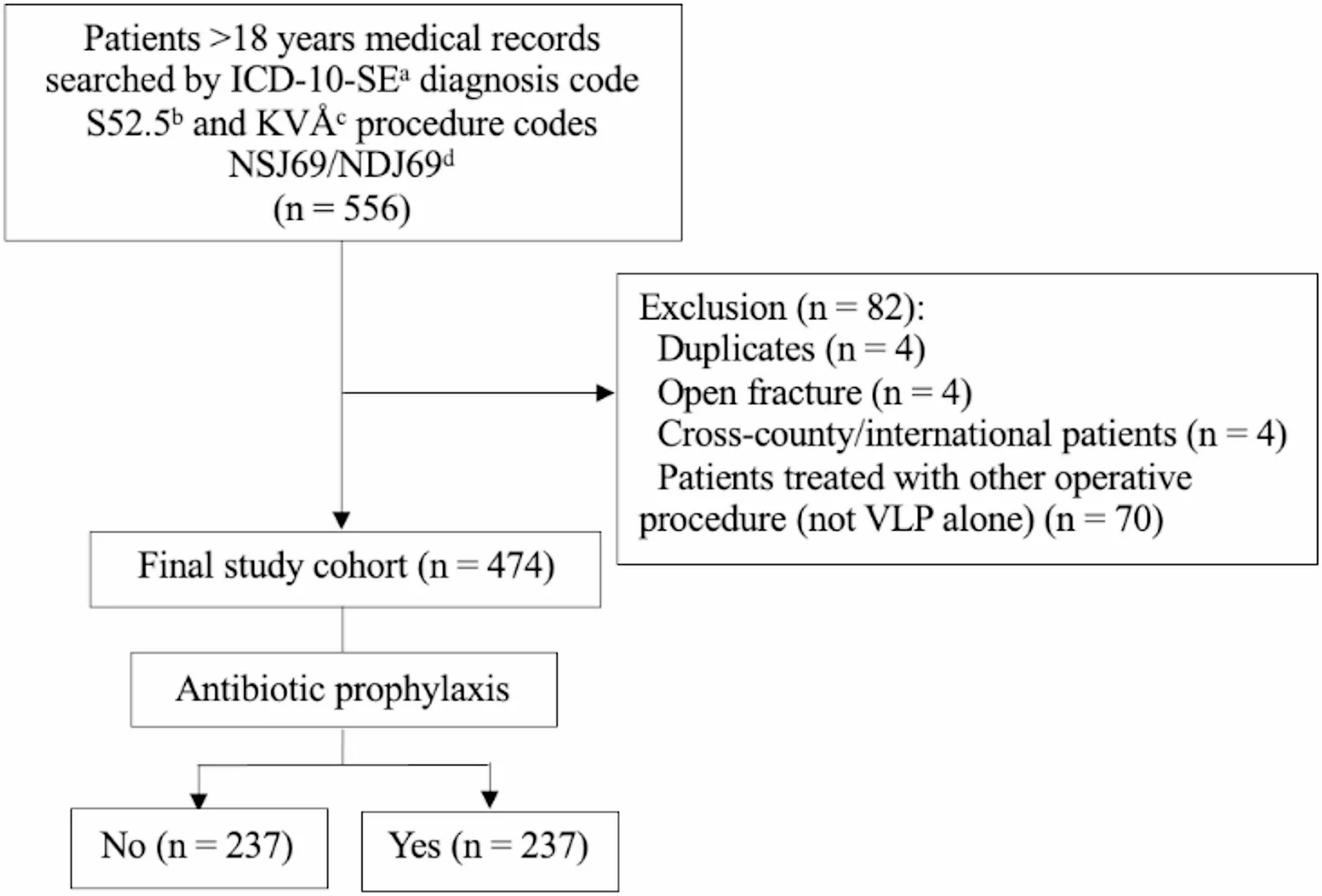
Flow chart of the patient selection process. ^a^International Statistical Classification of Diseases version 10. ^b^Fractures of lower end of radius. ^c^National procedure coding system (Classification of Health Care Measures, KVÅ). ^d^Fracture surgery using plate and screws of forearm/wrist

A flowchart of patient selection is shown in Fig. 1

**Table 1:** Inclusion and exclusion criteria

Inclusion	Exclusion
Age > 18 years	Non-operative cases
Operative treated closed DRFs with VLP	Treated with additional osteosynthesis or other operative treatment (e.g. carpal tunnel release)
Treated at regional hospital between January 2022 and December 2024	Open fractures
	Cross-county/international patients

DRF: Distal radius fracture; VLP: Volar locking plate

### Exposure

Patients were categorized according to whether perioperative antibiotic prophylaxis was administered as part of routine clinical care. Antibiotic prophylaxis, when administered, consisted of a single intravenous dose of flucloxacillin 2 g given 30–60 min before skin incision. In patients with documented penicillin allergy, clindamycin 600 mg intravenously was used, administered within the same time frame prior to incision.

### Outcomes

The primary outcome was postoperative surgical site infection within one year following surgery. Infections were classified as superficial or deep based on clinical and microbiological findings. Postoperative infections were identified through systematic review of electronic medical records, including outpatient visits, emergency department consultations, readmissions, reoperations, and microbiological reports within one year of surgery. Infection was defined clinically by treating physicians based on local signs of infection with or without microbiological confirmation. No routine surveillance cultures were performed.

Other variables included operative time, surgeon’s level of expertise and follow-up duration.

### Surgeon’s level of expertise

Surgeon expertise was graded according to Tang and Giddins, with level 1 representing trainees or non-specialists, level 2 specialists with limited experience, level 3 experienced specialists, and level 4 highly experienced specialists [16].

### Statistical analysis

Statistical analyses were performed using IBM SPSS Statistics (version 30.0; IBM Corp., Armonk, NY, USA). Categorical variables were compared using the chi-square test. Continuous variables were analysed using independent samples t-tests, while ordinal or non-normally distributed variables were analysed using the Mann–Whitney U test. Statistical significance was defined as a two-sided p-value < 0.05. Due to the very low number of infection events, formal comparative analysis of postoperative infection rates between prophylaxis groups was not performed. Accordingly, analyses of infection outcomes were descriptive in nature. Continuous variables are presented as mean (standard deviation) or median (interquartile range) as appropriate, and categorical variables as frequencies and percentages.

### Ethical considerations

The study was approved by the Swedish Ethical Review Authority (Dnr 2025-08625-01) and conducted in accordance with the Declaration of Helsinki.

## Results

### Patient characteristics

A total of 474 consecutive patients were included. Of these, 237 received perioperative antibiotic prophylaxis and 237 did not, reflecting routine clinical practice rather than predefined treatment allocation. Baseline characteristics are presented in Table 2. Differences were observed between prophylaxis groups with respect to ASA classification and surgeon expertise, reflecting variation in clinical practice.

**Table 2:** Characteristics of the study group

		Antibiotic prophylaxis			
		No (*N* = 237)	Yes (*N* = 237)	Total (*N* = 474)	*p*-value
Age	Median (range)	64 (18–90)	66 (19–89)		0.560^a^
Sex	Female	201 (84.8%)	190 (80.2%)	391 (82.5%)	0.184^b^
Comorbidity	Smoker	19 (8.0%)	23 (9.7%)	42 (8.9%)	0.518^b^
	Immunosuppressive condition	20 (8.4%)	18 (7.6%)	38 (8.0%)	0.735^b^
	Diabetes	12 (5.1%)	21 (8.9%)	33 (7.0%)	0.104^b^
ASA-class^*^	1	80 (33.8%)	60 (25.3%)	140 (29.5%)	0.006^a^
	2	135 (57.0%)	136 (57.4%)	271 (57.2%)	
	3	22 (9.3%)	39 (16.5%)	61 (12.9%)	
	4	-	2 (0.8%)	2 (0.4%)	

SD: Standard deviation

^a^Mann-Whitney U test was used for calculation of p-value

^b^Chi-square test was used for calculation of p-value

^*^American Society of Anesthesiologists (ASA) physical status classification system; 1: a healthy patient; 2: a patient with systemic disease that is mild; 3: a patient with a systemic disease that is severe but not life-threatening; 4: a patient with “severe systemic disease that is a constant threat to life”; 5: a comatose patient who is “not expected to survive without operation”; 6: a brain dead patient whose organs are being procured for transplantation into another patient

### Primary outcome

One postoperative infection occurred, corresponding to an overall infection proportion of 0.2% (95% CI 0.0–1.2%). The infection proportion was 0.4% (1/237; 95% CI 0.0–2.3%) in the non-prophylaxis group and 0% (0/237; 95% CI 0–1.5%) in the prophylaxis group. The infection was superficial, occurred in a patient who did not receive antibiotic prophylaxis and was successfully treated with oral antibiotics. No deep infections were observed. Details are presented in Table 3.

**Table 3:** Characteristics of the patient who had a postoperative infection

Antibiotic prophylaxis	Age	Sex	Comorbidity	ASA	Surgeon’s level of expertise	Type of infection	Culture findings	Antibiotic treatment	Reoperation required
No	44	Male	None	1	2	Superficial	*S. aureus*	Flucloxacillin 1 g x 3, 10 days	No

ASA: American Society of Anesthesiologists physical status classification; *S. aureus*: *Staphylococcus aureus*

### Patient and surgical characteristics

Patient, surgical, and surgeon-related characteristics are presented in Table 4. No deep infections or infection-related reoperations were identified during the one-year follow-up period.

**Table 4:** Independent variables

		Antibiotic prophylaxis			
		No (*N* = 237)	Yes (*N* = 237)	Total (*N* = 474)	*p*-value
Injury to operation, days	Median (range)	8 (1–21)	8 (0–22)		0.398^a^
Surgeon’s level of expertise^*^	1	19 (8.0%)	32 (13.5%)	51 (10.8%)	< 0.001^a^
	2	130 (54.9%)	155 (65.4%)	285 (60.1%)	
	3	87 (36.7%)	47 (19.8%)	134 (28.3%)	
	4	1 (0.4%)	3 (1.3%)	4 (0.8%)	
Operation time, minutes	Mean (SD)	90.8 (25.8)	93.9 (28.7)		0.211^b^
	Median	87	89		
	Range	40–215	41–228		
LOS, days	0	228 (96.2%)	204 (86.1%)	432 (91.1%)	< 0.001^a^
	≥ 1	9 (3.8%)	33 (13.9%)	42 (8.9%)	
Follow-up period, months	Mean (range)	29.3 (10–46)	26.8 (6–46)		0.011^b^

SD: Standard deviation; LOS: Length of stay

^a^Mann-Whitney U test was used for calculation of p-value

^b^Independent t-test was used for calculation of p-value

^*^Level of expertise of the surgeons based on Tang and Giddins criteria

## Discussion

The principal findings of this study were the very low postoperative infection rate following volar locking plate fixation of closed distal radius fractures and the substantial variation in perioperative antibiotic prophylaxis practice observed within a single institution. Only one superficial postoperative infection occurred among 474 consecutive patients, and no deep infections were identified during one year of follow-up.

The infection rate observed in the present cohort is consistent with previously reported rates following VLP fixation, which range from approximately 0.1% to 4%[17.18]. The absence of deep infections in the present cohort further supports the notion that this procedure carries a low baseline risk of serious infection.

The present study was not designed or powered to evaluate the effectiveness of perioperative antibiotic prophylaxis. Because only a single postoperative infection was observed, any comparison between prophylaxis groups would be unreliable and potentially misleading. Accordingly, the findings should be interpreted as descriptive rather than comparative. Nevertheless, the low infection rate observed aligns with previous studies reporting infection rates between 0.1% and 4% following volar locking plate fixation of distal radius fractures [13–15, 17, 18]. Importantly, the absence of deep infections in our cohort supports the notion that serious postoperative infections are uncommon in this setting, and that superficial infections, when they occur, are generally mild and manageable with oral antibiotics.

A notable finding was the substantial variation in antibiotic prophylaxis practice among surgeons within the same institution. A higher proportion of patients with greater comorbidity, reflected by ASA class ≥ 3, received prophylaxis, whereas healthier patients (ASA 1) more often did not. Higher ASA classification has previously been associated with increased postoperative infection and wound complication risk following distal radius fracture surgery, including VLP fixation. This may partly explain why surgeons in the present cohort more frequently administered prophylaxis to patients perceived to be at greater perioperative risk; however, the available evidence does not establish that selective antibiotic prophylaxis based on ASA classification reduces infection risk [18, 19]. Similarly, less experienced surgeons tended to administer prophylaxis more frequently than those with higher expertise levels. These observations suggest that prophylaxis decisions may be influenced by individual surgeon judgment, perceived patient risk, and perhaps concerns about complications or professional expectations, rather than clear evidence-based guidelines. Comparable trends have been reported in previous studies examining hand and wrist fracture surgery, highlighting the variability in clinical practice when high-quality evidence is limited [14, 15].

While patient-specific risk factors such as male sex, smoking, and comorbidities have been associated with increased risk of postoperative infection in distal radius fracture surgery, these factors did not appear to influence infection outcomes in our cohort, likely due to the overall rarity of infection [18]. The single postoperative infection occurred in a young, otherwise healthy male and was mild, requiring only oral antibiotics. This finding reinforces the low baseline risk of infection in this population and highlights that even in the absence of prophylaxis, severe complications are uncommon.

The findings should primarily be interpreted as descriptive data from a contemporary cohort undergoing volar locking plate fixation for closed distal radius fractures. The very low infection burden observed in this study is consistent with previous reports and provides additional information regarding current clinical practice in a common orthopaedic procedure. Reducing prophylactic antibiotic use could also improve cost-effectiveness, reduce the risk of adverse drug reactions, and help mitigate the broader public health concern of antibiotic resistance [17]. Although the contribution of a single perioperative antibiotic dose to antimicrobial resistance is likely to be limited, judicious use of prophylactic antibiotics remains a fundamental principle of antimicrobial stewardship according to current international guidelines [9]. Although no serious antibiotic-related adverse events were identified during the review of the medical records, adverse drug reactions were not a predefined study outcome and may therefore have been underreported. At our institution, the acquisition cost of a single prophylactic intravenous dose is approximately 206 SEK for flucloxacillin and 59 SEK for clindamycin. According to the Swedish Fracture Register annual reports, approximately 14,000 volar locking plate fixations for distal radius fractures were performed in Sweden over the three consecutive years corresponding to the present study period (2022–2024) [20]. Based on the institutional acquisition costs reported above, routine perioperative antibiotic prophylaxis would be associated with an estimated direct antibiotic acquisition cost of approximately 2.8 million SEK nationwide over the same three-year period. This estimate is intended solely to illustrate the potential population-level implications of routine prophylaxis and does not represent a formal health-economic analysis. These data may contribute to future discussions regarding antibiotic stewardship, although the effectiveness of prophylaxis cannot be determined from the present study.

Our study has several limitations that should be considered when interpreting the results. The retrospective design may have led to under-reporting of minor infections treated outside our institution. However, within the national healthcare system, postoperative wound care and routine follow-up after distal radius fracture surgery are typically managed at the treating clinic, which may reduce the risk of missed postoperative infections. Differences in baseline characteristics between groups, particularly ASA class and surgeon experience, limit causal inference regarding the impact of prophylaxis. Although diabetes, smoking status, immunosuppressive treatment and ASA classification were available, other individual comorbidities, such as chronic kidney disease or liver disease, were not collected separately and therefore could not be evaluated. Additionally, the small number of infection events precludes formal statistical comparisons. Consequently, the study cannot determine whether perioperative antibiotic prophylaxis influences postoperative infection risk. Patients with higher ASA classification were more likely to receive prophylaxis. Given the extremely low event probability, we were unable to account for potential confounding by indication. It remains possible that patients perceived to be at higher risk of infection may derive greater benefit from prophylaxis. Given the very low baseline infection proportion observed, detecting a clinically meaningful difference between groups would require a substantially larger sample size. For example, assuming an infection proportion below 0.5%, several thousand patients would likely be needed to demonstrate a statistically reliable difference. This underscores the challenge of studying rare adverse events and suggests that the question may remain difficult to answer definitively without large multicenter collaboration. Nonetheless, the relatively large cohort, real-world setting, and inclusion of all eligible cases over a three-year period strengthen the generalizability of our findings.

Future multicentre studies with substantially larger sample sizes are needed to better characterize postoperative infection risk and evaluate the potential role of perioperative antibiotic prophylaxis in distal radius fracture surgery. Such studies could further clarify which patient or surgical factors, if any, modify infection risk and inform evidence-based guidelines for prophylaxis use. In our cohort, postoperative infection following volar locking plate fixation of closed distal radius fractures was rare. While the study cannot determine the effectiveness of antibiotic prophylaxis, it provides descriptive data from a single-center cohort with complete one-year follow-up and equally sized comparison groups. These findings may inform future studies evaluating postoperative infection risk and perioperative antibiotic prophylaxis in distal radius fracture surgery.

## Conclusions

Postoperative infection following volar locking plate fixation of closed distal radius fractures was rare in this contemporary single-center cohort, with only one superficial infection identified among 474 consecutive patients. Considerable variation in perioperative antibiotic prophylaxis practice was observed despite uniformly low infection rates. While the study was not powered to evaluate the effectiveness of antibiotic prophylaxis, it provides descriptive data on postoperative infection burden and current prophylaxis practice following volar locking plate fixation of closed distal radius fractures.

## Data Availability

The datasets used and/or analysed during the current study are available from the corresponding author on reasonable request.
